# Factors influencing infection and transmission of *Anopheles gambiae* densovirus (AgDNV) in mosquitoes

**DOI:** 10.7717/peerj.2691

**Published:** 2016-11-09

**Authors:** Tapan K. Barik, Yasutsugu Suzuki, Jason L. Rasgon

**Affiliations:** 1 Applied Entomology Laboratory, Post Graduate Department of Zoology, Berhampur University, Berhampur, Odisha, India; 2 Department of Entomology, Pennsylvania State University, University Park, PA, United States; 3 Department of Virology, Institute Pasteur, Paris, France; 4 Center for Infectious Disease Dynamics, Pennsylvania State University, University Park, PA, United States; 5 The Huck Institutes of the Life Sciences, Pennsylvania State University, University Park, PA, United States

**Keywords:** Malaria, Paratransgenesis, Gene transduction, Sex-specific differences, Vector-borne disease, Disease control

## Abstract

*Anopheles gambiae* densovirus (AgDNV) is a potential microbial agent for paratransgenesis and gene transduction in *An. gambiae*, the major vector of human malaria in sub-Saharan Africa. Understanding the interaction between AgDNV and *An. gambiae* is critical for using AgDNV in a basic and applied manner for *Anopheles* gene manipulation. Here, we tested the effects of mosquito age, sex, blood feeding status, and potential for horizontal transmission using an enhanced green fluorescent protein (EGFP) reporter AgDNV system. Neither mosquito age at infection nor feeding regime affected viral titers. Female mosquitoes were more permissive to viral infection than males. Despite low viral titers, infected males were able to venereally transmit virus to females during mating, where the virus was localized with the transferred sperm in the spermathecae. These findings will be useful for designing AgDNV-based strategies to manipulate *Anopheles gambiae*.

## Introduction

Mosquitoes in the genus *Anopheles* are the only arthropods capable of transmitting the *Plasmodium* parasites that cause human malaria, which infects over 200 million people and kills nearly one million people globally each year (Enayati & Hemingway, 2010). *Anopheles gambiae* is the most efficient human malaria vector in sub-Saharan Africa (Mendes et al., 2008). Paratransgenesis, the genetic manipulation of insect symbiotic microorganisms to inhibit pathogen replication in the hosts, has been proposed as a novel strategy to control mosquito-borne diseases such as malaria (Ren, Hoiczyk & Rasgon, 2008; Rasgon, 2011; Wang & Jacobs-Lorena, 2013). Previous studies demonstrated that paratransgenesis is able to dramatically diminish *Plasmodium* parasite density in *An. gambiae* (Fang et al., 2011; Wang et al., 2012). In addition to pathogen restriction in individual hosts, methods to spread transgenic symbionts into natural mosquito populations are critical for the application of this technology (Rasgon, 2008). Understanding basic interactions between the paratransgenic agent and its host arthropod is critical to develop successful paratransgenesis-based approaches for disease control.

Densonucleosis viruses (densoviruses (DNVs)) are non-enveloped, single-stranded parvoviruses that have been identified from many invertebrate taxa, including various mosquito species (Carlson, Suchman & Buchatsky, 2006). Their stability, narrow host range, and pathogenicity make DNVs attractive as bio-pesticides (Carlson, Suchman & Buchatsky, 2006; Suzuki et al., 2015). In mosquitoes, *Aedes aegypti* densovirus (AeDNV) has been intensively studied as a transducing vector and biocontrol agent against *Aedes* mosquitoes (Allen-Miura et al., 1999; Ledermann et al., 2004; Gu et al., 2010). We recently reported that *An. gambiae* DNV (AgDNV) can efficiently transduce exogenous genes in *An. gambiae* without serious pathogenic effects; DNV-infected mosquitoes had similar immature and adult lifespan compared to uninfected controls, and infection by the virus had minimal effects on host gene expression (Ren, Hoiczyk & Rasgon, 2008; Ren & Rasgon, 2010; Ren et al., 2014; Suzuki et al., 2014; Suzuki et al., 2015). These lack of viral-induced pathogenic effects make AgDNV a potentially useful as a viral paratransgenic agent for malaria control, as the agent itself is unlikely to be eliminated from the population due to negative selection. However, nothing is currently known about how basic components of mosquito biology (such as aging, feeding and mating) affect AgDNV infection dynamics in the mosquito, which could potentially affect disease control efforts. Here, we tested the effects of mosquito age, sex, blood feeding status, and potential for horizontal transmission to investigate the potential for these factors to help or hinder AgDNV spread in natural populations.

## Methods

### Insect cell culture and mosquito rearing

Uninfected *An. gambiae* Moss55 cells were cultured in Schneider’s medium (Sigma) supplemented with 10% fetal bovine serum (FBS) at 28 °C. *An. gambiae* mosquitoes (Keele strain) were reared at 28 °C and 80% relative humidity with 12:12 h light:dark photoperiod and offered 10% sugar at all times. Female adult mosquitoes were blood fed on commercially purchased expired human blood through artificial feeding system. Larvae were fed with tetramin fish food. During experiments, mosquitoes were reared on sugar except in cases where the effect of bloodfeeding was being specifically examined.

### Production of recombinant AgDNV samples

Recombinant viruses were produced by co-transfecting the recombinant AgDNV plasmid pUTRAcGFP (Suzuki et al., 2014) and the helper plasmid pBAgα (Ren, Hoiczyk & Rasgon, 2008) at a ratio of 2:1 using Lipofectamine LTX reagent (Life Technologies) according to the manufacturer’s protocol in 70% confluent Moss55 cells. After three days of incubation, cells were harvested and suspended in phosphate buffered saline (PBS). The cell suspension was subjected to freezing-thawing three times and cell debris removed by centrifugation. The supernatant was used as the viral inoculum for experiments.

### Densovirus DNA quantification

For quantifying DNV levels in inoculum, samples were subjected to TURBO DNase (Ambion) treatment to digest any residual plasmid DNAs prior to DNA extraction. For quantification of DNV in mosquitoes, total DNA was extracted from DNV-injected mosquitoes or tissues using DNEasy kits (Qiagen) according to the manufacturer’s protocol. Quantitative polymerase chain reaction (PCR) (qPCR) was performed (either absolute titers using a standard curve or normalizing to the mosquito S7 gene depending on the experiment) using Quantitect SYBR Green Kit (Qiagen) on a RotorGene system (Qiagen) with previously described primers (Suzuki et al., 2014).

### AgDNV infection of *An. gambiae* mosquitoes

For mosquito infections, 20 adult mosquitoes per treatment were injected intrathoracically with virus as previously described at 10^6^ and 10^7^ viral genome equivalents per ml (vge/ml) as described in the text (Suzuki et al., 2014; Suzuki et al., 2015). For testing the effect of mosquito age on infection, mosquitoes were infected at 1 and 7 days post-emergence, and were held for an additional seven days before assaying them for virus. For testing the effect of mosquito feeding on infection, we controlled for the effect of mosquito age by infecting mosquitoes in all three treatments seven days post-emergence, and held them for an additional seven days before being assayed for infection. Mosquitoes in treatment 1 were maintained only on sugar for the entire time. Mosquitoes in treatment 2 were bloodfed three days post-injection (10 days post-emergence). Mosquitoes in treatment 3 were bloodfed one day prior to virus injection (six days post-emergence). For monitoring viral in vivo replication, mosquitoes were infected five days post-emergence and sampled three, seven and 12 days post-injection.

### Microscopy analysis of EGFP expression

After microinjection, five individual mosquitoes/sex/treatment were randomly collected at the time points listed in the text and fluorescence observed using an Olympus BX-41 epifluorescent microscope. Images were processed using Picture Frame software (Olympus). After microscopy analysis, mosquitoes were stored at −80 °C until DNA was extracted for qPCR analysis.

### Venereal transmission

Male and female mosquitoes were separated at the pupal stage and kept in different cages. The 5-day-old males were injected with vUTRAcGFP or media as described above. Three days post-injection, males were allowed to mate with uninfected females for seven days. After mating, females were collected, examined for EGFP by microscopy, and their spermathecae dissected into PBS. Viral levels in the spermathecae and carcasses were assayed by qPCR.

### Statistical analysis

Experiments with paired treatments were analyzed by t-tests. Experiments with > 2 treatments were analyzed by ordinary one-way or two-way analysis of variance (ANOVA) as appropriate with Bonferroni’s correction for multiple tests. Prior to analysis, data were tested for adherence to normality using the Shapiro-Wilk normality test. Data that did not conform to normality assumptions were log transformed prior to analysis. All analyses were conducted using GraphPad Prism v.7.

## Results

### Mosquito age and AgDNV replication

**Figure 1:**
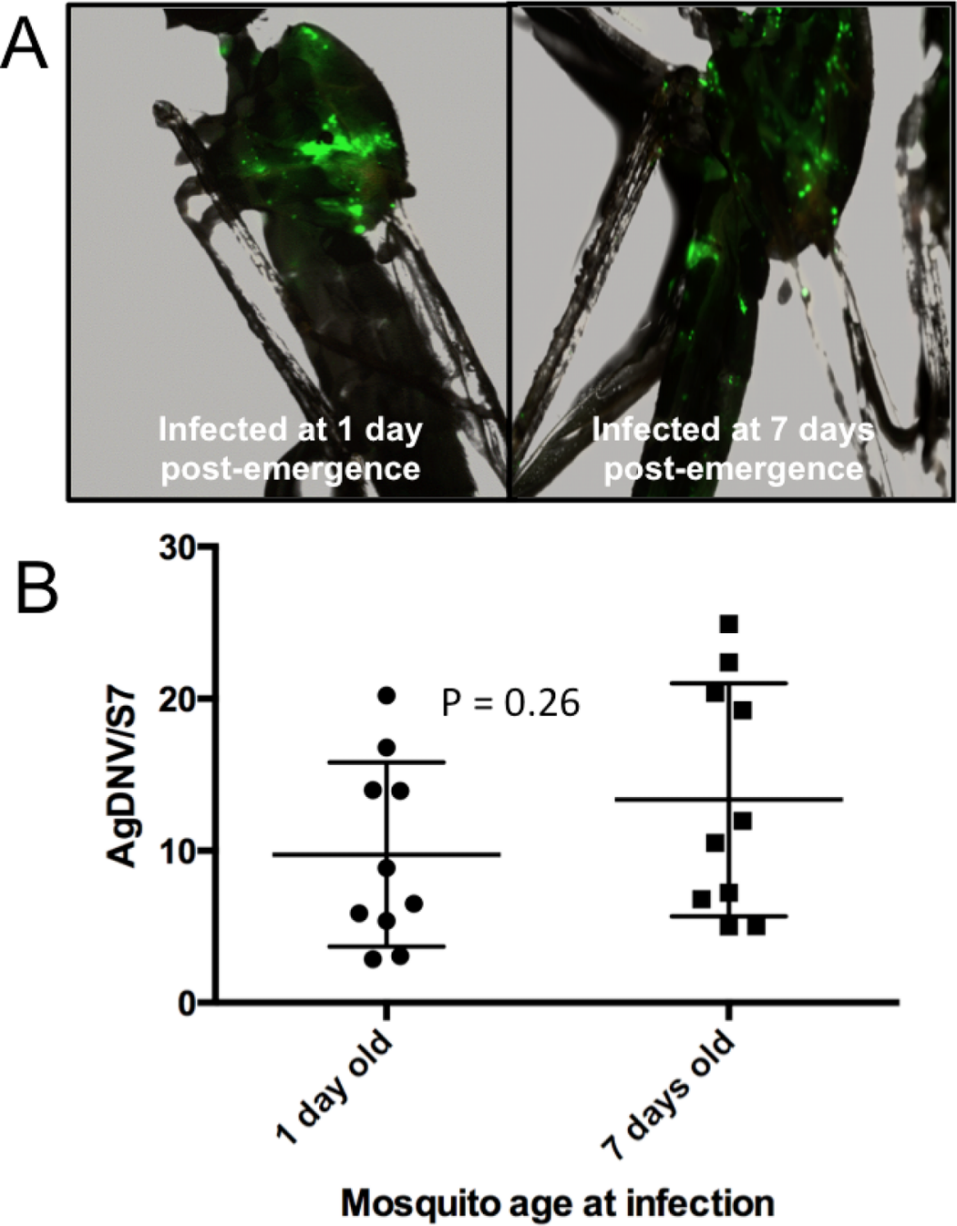
Effect of mosquito age at infection on AgDNV replication. Mosquitoes were infected at either one or seven days post-emergence. (A) EGFP expression. (B) Quantitation of AgDNV by qPCR. Infection levels between treatments are not statistically different (T-test).

Adult female mosquitoes (one or seven days post emergence) were intrathoracically inoculated with 10^7^ viral genome equivalents (vge) of EGFP-transducing AgDNV (vUTRAcGFP) (Suzuki et al., 2014). Seven days post infection, mosquitoes were collected and examine for EGFP expression by fluorescent microscope. Mosquitoes infected at one and seven days post emergence exhibited qualitatively similar levels of EGFP expression (Fig. 1A), and quantitatively similar levels of viral genomes by qPCR (T = 1.17, df = 18, P = 0.26) (Fig. 1B).

### Mosquito blood feeding and AgDNV replication

**Figure 2:**
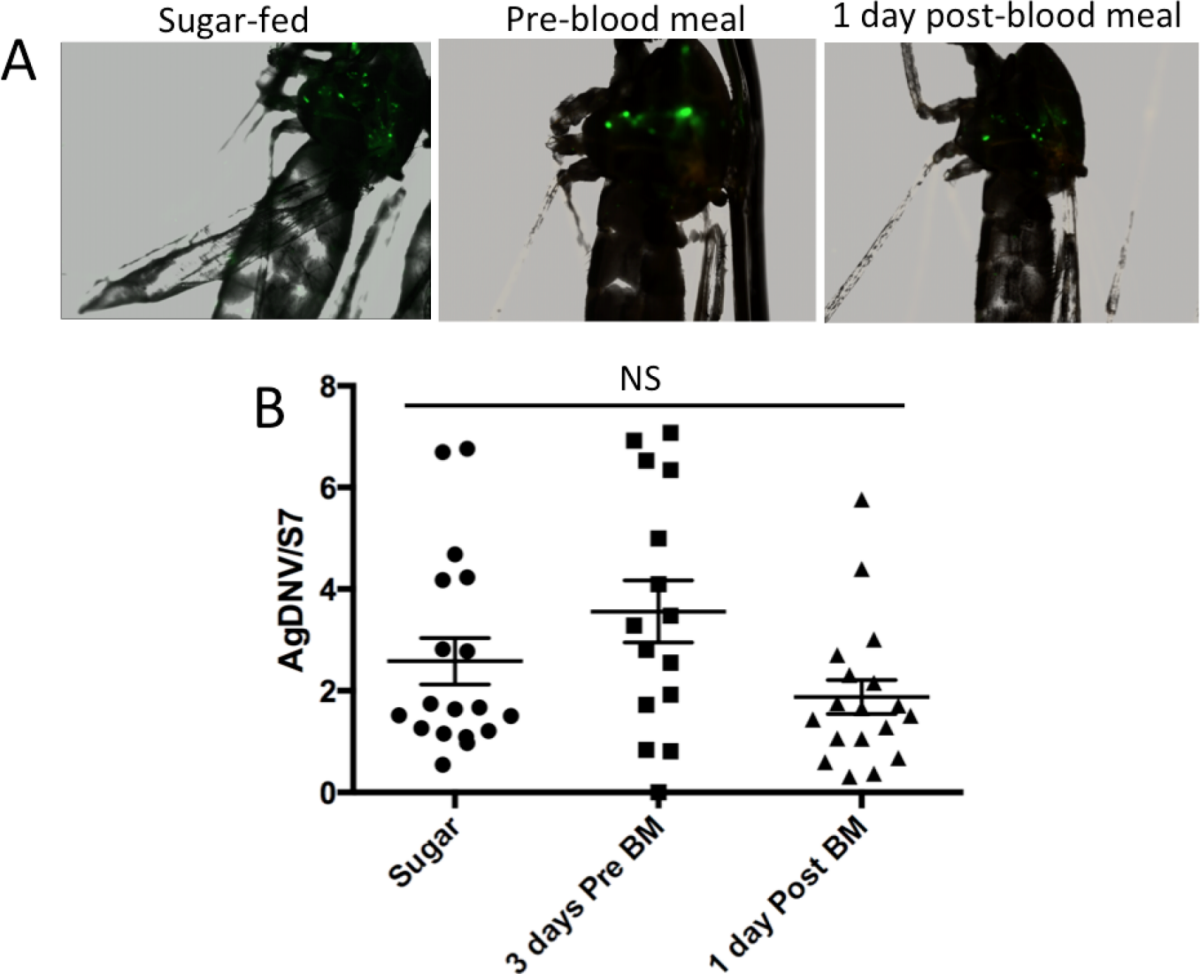
Effect of mosquito feeding on AgDNV replication. Mosquitoes were either sugar-fed, infected three days pre-blood meal, or infected one day post blood-meal. (A) EGFP expression. (B) Quantitation of AgDNV by qPCR. Infection levels between treatments are not statistically different (ANOVA).

Adult females were injected with 10^6^ vUTRAcGFP at three days before blood feeding, one day after blood feeding, or were only sugar-fed. Seven days post infection, mosquitoes were collected and examine for EGFP expression by fluorescent microscope. All three treatments exhibited qualitatively similar levels of EGFP expression (Fig. 2A). We then confirmed fluorescence results by qPCR to measure viral titers. Data were log transformed and analyzed by one-way ANOVA. Infection titers between treatments were not statistically different (F_2, 48_ = 0.5136, P = 0.6).

### *An. gambiae* females are more permissive to AgDNV than males

**Figure 3:**
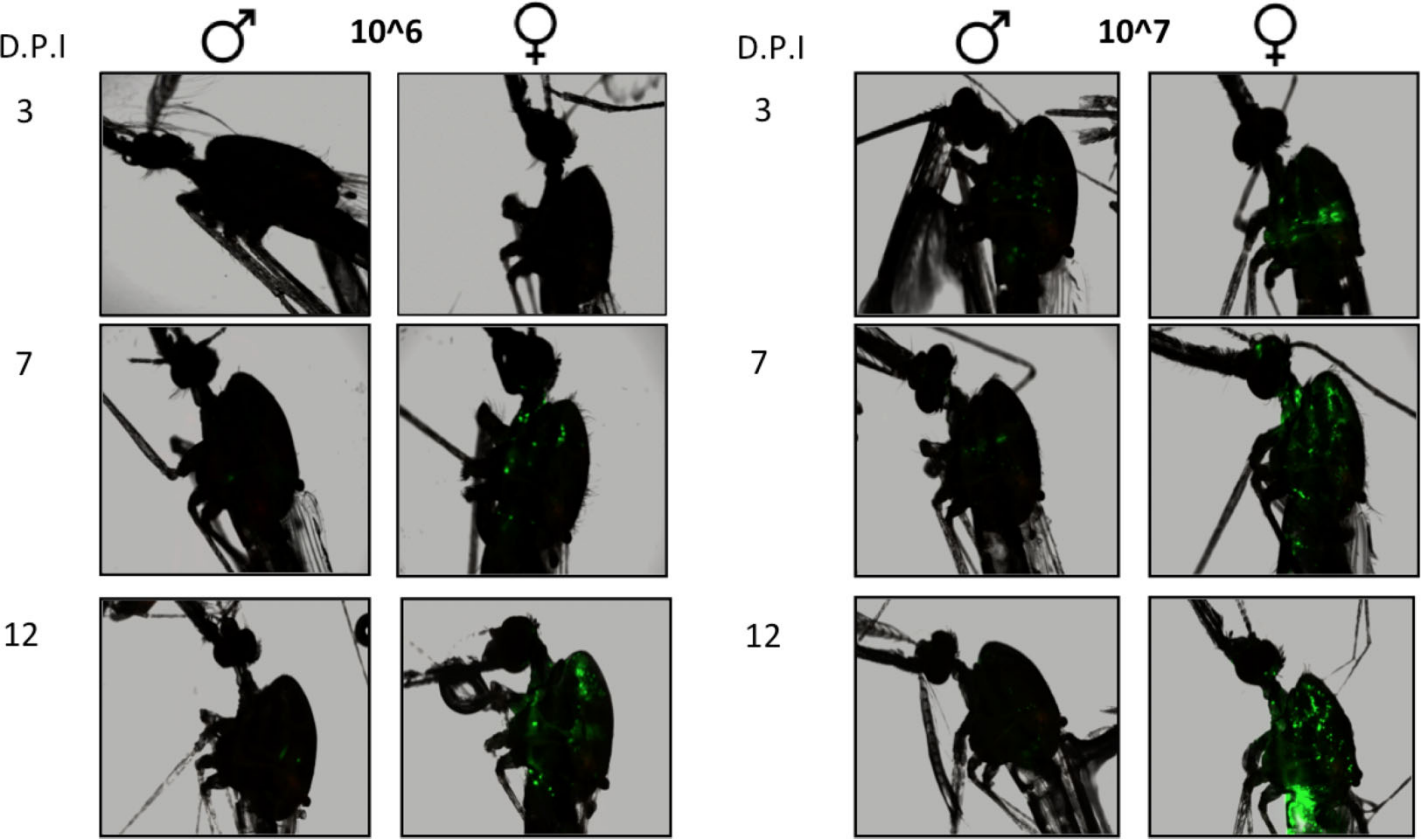
Effect of sex, time, and viral dose on AgDNV replication, assessed by EGFP fluorescence. At all time points and dosages, females exhibit greater EGFP expression compared to males. D.P.I., days post-injection.

**Figure 4:**
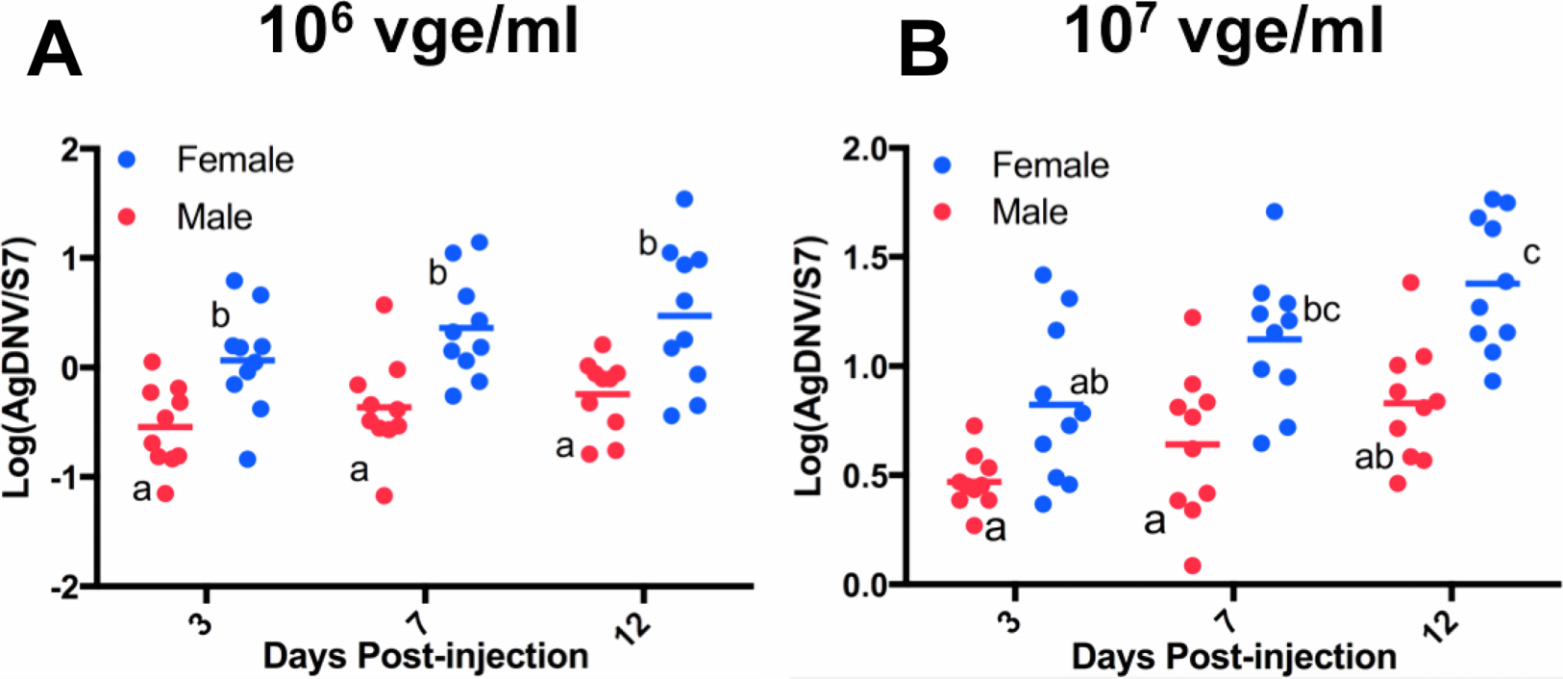
Effect of sex, time, and viral dose on AgDNV replication, assessed by qPCR. At all time points and dosages, females exhibit greater AgDNV titers compared to males. Data were analyzed by ANOVA. Treatments with different lowercase letters denote statistical significance (P < 0.05).

The 5-day-old female and male mosquitoes were injected with 10^6^ or 10^7^ vUTRAcGFP and assayed at three, seven and 12 days post-injection to monitor EGFP expression. Female mosquitoes showed an increase in EGFP fluorescence in a dose- and time-dependent manner (Fig. 3). Fluorescence results were independently confirmed by qPCR (Fig. 4). Data were log transformed and analyzed by ordinary 2-way ANOVA. At 10^6^ vge/ml, at all time points females exhibited greater viral titers compared to males. (F_1, 54_ = 31.73, P < 0.0001). There was a trend toward increased viral titer in females over time, but this was not significant (F_2, 54_ = 2.897, P = 0.06). At 10^7^ vge/ml, viral titers varied significantly by both sex (F_1, 54_ = 36.34, P < 0.0001) and time (F_2, 54_ = 11.86, P < 0.0001) (Fig. 4). The interaction terms were not significant.

### AgDNV can be venereally transmitted from males to females

**Figure 5:**
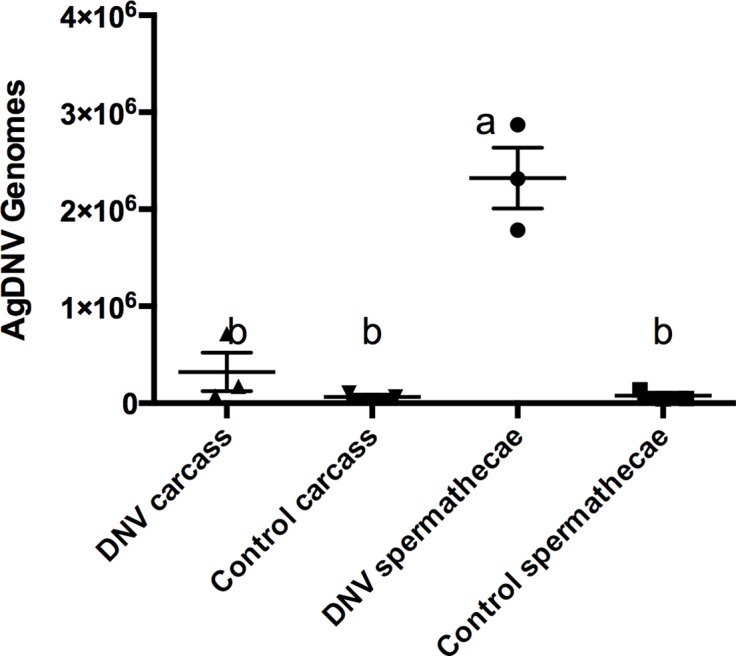
AgDNV can be venereally transmitted from males to females, and is present in the spermathecae. Data were analyzed by ANOVA.

Although females were more permissive to AgDNV infection than males, males did get infected. We therefore investigated the possibility that males could transfer AgDNV to females during mating. Male mosquitoes were injected with 10^7^ vUTRAcGFP or media as a control. Three days-post injection, males were mated to uninfected females. Mosquitoes were allowed to mate for seven days, after which females were collected to assay for virus. EGFP was not observed in females mated to AgDNV-infected males (data not shown). Spermathecae of mated females were dissected and the spermathecae and carcasses assayed for virus by qPCR. Data were analyzed by ordinary one-way ANOVA. Viral titers were significantly higher in spermethecae of females mated to infected males (F_3, 8_ = 34.04, P < 0.0001) compared to the spermethecae of females mated to uninfected males, or the carcasses of females mated to either male type (Fig. 5).

## Discussion

Paratransgenesis, the genetic manipulation of arthropod symbiotic microorganisms, is one potential strategy to control mosquito-borne diseases (Ren, Hoiczyk & Rasgon, 2008; Ren & Rasgon, 2010; Rasgon, 2011; Fang et al., 2011; Wang et al., 2012; Wang & Jacobs-Lorena, 2013). Interactions between mosquito hosts and their microbes need to be well-understood for successful design of paratransgenic control methods. Here, we investigated the effects of mosquito age, feeding status and sex on AgDNV infection.

Neither timing of infection after emergence or mosquito feeding significantly affected virus replication. However, female mosquitoes were more permissive to virus infection than males at multiple viral titers. We previously showed that the mosquito fat body is a main location of viral replication (Ren, Hoiczyk & Rasgon, 2008; Suzuki et al., 2014; Suzuki et al., 2015). Females are larger and have greater abundance of this tissue, and differences in infection levels may simply be due to differences in amounts of permissive tissue between sexes.

Although males were less permissive to virus infection than females, they were able to transmit the virus venereally to their mates. However, EGFP expression and qPCR indicated that transferred virus was restricted to the female spermatheca and did not disseminate in the female. This is similar to *Ae. albopictus* parvovirus (AaPV), which was venereally transmitted at a low rate without detectable dissemination (Barreau, Jousset & Bergoin, 1997). As AgDNV is transmitted vertically from mother to offspring (Ren, Hoiczyk & Rasgon, 2008), future studies should test the possibility that venereally transferred virus may be able to infect offspring during fertilization, as it may facilitate the spread of the virus into natural populations during a paratransgenic control release.

## Supplemental Information

10.7717/peerj.2691/supp-1 Supplemental Information 1 Raw data for Figs. 1B, 2B, 4 and 5.

## Additional Information and Declarations

### Data Deposition

The following information was supplied regarding data availability: All raw data are presented in the relevant figures.
